# A new flatworm species of *Temnocephala* (Rhabdocoela, Temnocephalidae) ectosymbiont on the freshwater crab *Valdivia serrata* (Decapoda, Trichodactylidae) from Amazonas, Colombia

**DOI:** 10.3897/zookeys.918.38201

**Published:** 2020-03-12

**Authors:** Carolina Lenis, Freddy Ruiz, Carlos Muskus, Antonio Marcilla, Imelda Vélez

**Affiliations:** 1 PECET. Programa de Estudio y Control de Enfermedades Tropicales. Facultad de Medicina, Universidad de Antioquia, Calle 62 No. 52-59, Medellín, Colombia Universidad de Antioquia Medellín Colombia; 2 Departamento de Farmacia y Tecnología Farmacéutica y Parasitología, Facultat de Farmacia, Universitat de València, 46100 Burjassot, Valencia, Spain Universitat de València València Spain

**Keywords:** Crustacea, Reserva Natural Tanimboca, taxonomy, *Temnocephala ivandarioi* sp. nov.

## Abstract

A new species of temnocephalan is described from the branchial chambers of *Valdivia serrata* in Colombia as *Temnocephala ivandarioi***sp. nov.** The most distinctive characters of the new species are in the cirrus and the epidermal ‘excretory’ syncytial plates. In the present study, the terminology to describe the cirrus of species of *Temnocephala* is updated. Comparison between the shape of the cirrus of the temnocephalans associated with trichodactylid crabs is also provided.

## Introduction

The genus *Temnocephala* Blanchard, 1849 includes 37 species of symbiotic freshwater rhabdocoels from the Neotropics, which are associated with a large variety of hosts including chelonians (3), mollusks (6), insects (7) and crustaceans (21) (Garcés et al. 2013, Martínez-Aquino et al. 2014, Arias-Pineda et al. 2015, Seixas et al. 2015a, Ponce de León and Volonterio 2018, Seixas et al. 2018). At present, only two species have been described from Colombia, *T. colombiensis* Garcés, Puerta, Tabares, Lenis & Velásquez, 2013, ectosymbiont of the gastropod *Pomacea* sp., and *T. icononcensis* Arias-Pineda, Damborenea & Castro, 2015, hosted by the pseudothelphusid crabs *Hypolobocera bouvieri* (Rathbun 1898), *Phallangothelphusa dispar* (Zimmer 1912) and *Strengeriana cajaensis* Campos & Rodríguez, 1993. In this paper, we describe and illustrate a new species of *Temnocephala* associated with the trichodactylid crab *Valdivia serrata* from the Colombian Amazonas.

## Materials and methods

Eleven specimens of *Valdivia serrata* White, 1847 (crab) were manually collected from the Reserva Natural Tanimboca, Leticia, Amazonas (4°07'39.8"S, 69°57'13.0"W), Colombia. The specimens were transported alive to the Programa de Estudio y Control de Enfermedades Tropicales (PECET) laboratory and identified using a decapod key (Campos 2014). Specimens of *Temnocephala* were removed from the branchial chambers of host crabs under a stereomicroscope, rinsed in saline solution, and preserved in cold alcohol-formalin-acetic acid (AFA) and 70% ethanol for morphological identification by light microscopy; or fixed in 2.5% glutaraldehyde for observation by Scanning Electron Microscopy (SEM). The temnocephalans were identified by focusing on the morphology of the reproductive complex, the shape of the epidermal excretory syncytial plates (DLSPs) and the deposit areas of the eggs in the host. Terminology to describe the cirrus of the temnocephalans was updated from Sewell et al. (2007), Seixas et al. (2011), Garcés et al. (2013) and Ponce de León et al. (2015). Morphology of the male and female reproductive system was studied by light microscopy in specimens mounted as permanent slides in Canada balsam, stained in Meyer’s paracarmine and Borax carmine. Samples were also observed by SEM to determine the shape and position of the egg filament, the fracture plane of the eggshell, the shape of the DLSPs and the relative position of the excretory pore. Measurements were in micrometres (µm) unless otherwise indicated; ranges were determined followed by the arithmetic mean, the standard deviation and the number of specimens measured for a given character (mean, standard deviation, n). Photomicrographs of the temnocephalans were taken with a Nikon Alphaphot YS-2 microscope. Drawings were made using a drawing tube Nikon 1.25X. Line drawings and photographic images were prepared using Inkscape 0.92. The SEM preparations were examined with a Hitachi S-4800 SEM at the Servicio de Microscopía, SCSIE, Universitat de València, Spain. Type specimens were deposited in the Colección Colombiana de Helmintos (CCH.116), Universidad de Antioquia, Medellín, Colombia.

## Taxonomy

**Phylum Platyhelminthes Minot, 1876**

**Order Rhabdocoela Ehrenberg, 1831**

**Suborder Dalytyphloplanida Willems, Wallberg, Jondelius, Littlewood, Backeljau, Schockaert & Artois, 2006**

**Infraorder Neotyphloplanida Willems, Wallberg, Jondelius, Littlewood, Backeljau, Schockaert & Artois, 2006**

**Parvorder Limnotyphloplanida Van Steenkiste, Tessens, Willems, Backeljau, Jondelius & Artois, 2013**

**Section Temnocephalida Blanchard, 1849**

**Superfamily Temnocephaloidea Baer, 1953**

**Family Temnocephalidae Monticelli, 1899**

### Genus *Temnocephala* Blanchard, 1849

#### Temnocephala ivandarioi sp. nov.

Taxon classificationAnimaliaRhabdocoelaTemnocephalidae

AB659D89-132A-5A69-AF77-FB117A431E6A

http://zoobank.org/E7284E9B-D311-46C3-B5CF-26CA544B15EF

Figs 1A –C , 2A –C

##### Type host.

**Figure 1.**
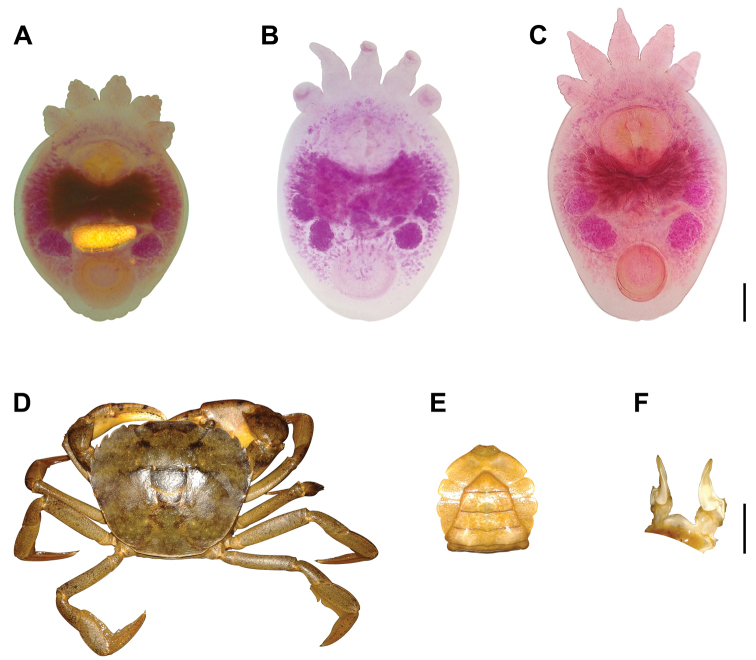
*Temnocephala ivandarioi* sp. nov. and *Valdivia serrata***A** paratype of *Temnocephala ivandarioi* sp. nov. showing an egg, ventral view **B** adult paratype stained in Meyer´s paracarmine **C** holotype stained in Borax carmine **D** male specimen of *V. serrata***E** abdomen **F** gonopods, lateral view. Scale bars: 200 µm (**A–C**); 10 mm (**D–F**).

*Valdivia serrata* White, 1847 (Fig. 1D–F).

##### Site of infection.

Branchial chambers.

##### Prevalence.

36% of the eleven hosts were infected.

##### Type locality.

Kilómetro 11, Reserva Natural Tanimboca, Leticia, Amazonas (4°07'39.8"S, 69°57'13.0"W), Colombia.

##### Type specimens.

Holotype: CCH.116 (159); Paratypes: CCH.116 (160).

##### Examined material.

10 whole mounted specimens; 5 stained in Meyer’s paracarmine; 5 stained in Borax carmine; 6 dissected cirrus; 2 samples observed by SEM, 5 unhatched eggs observed by SEM.

##### Description.

**Figure 2.**
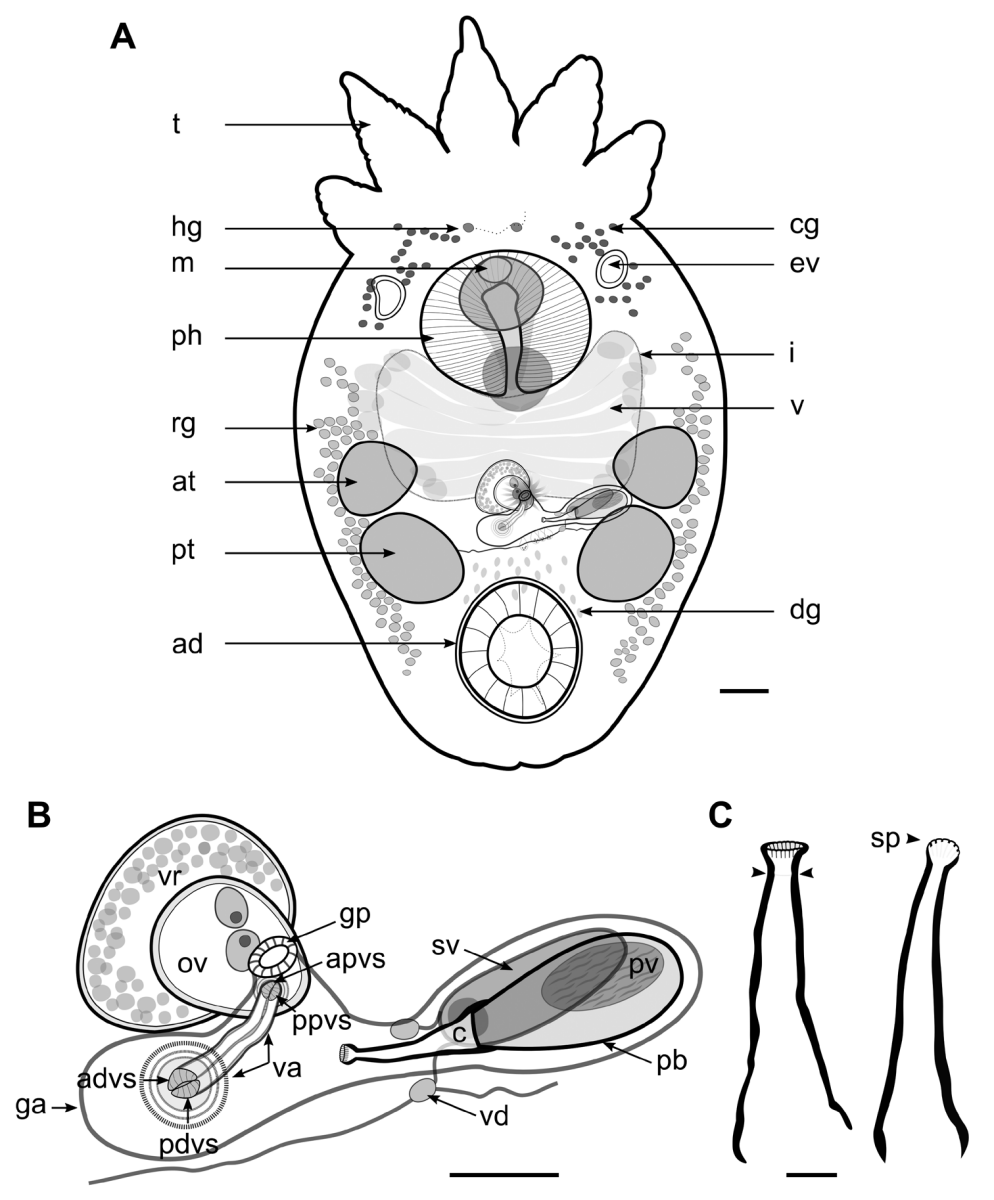
*Temnocephala ivandarioi* sp. nov. **A** adult specimen diagram, showing adhesive disk (ad), anterior testes (at), cyanophilous glands (cg), disk glands (dg), excretory vesicle (ev), Haswell´s glands (hg), intestinal sac (i), mouth (m), pharynx (ph), posterior testes (pt), rhabditogenic glands (rg), tentacles (t), and vitellarium (v) **B** reproductive system, showing female reproductive complex: anterior portion of the distal vaginal sphincter (advs), anterior portion of the proximal vaginal sphincter (apvs), genital atrium (ga), genital pore (gp), posterior portion of the distal vaginal sphincter (pdvs), posterior portion of the proximal vaginal sphincter (ppvs), ovary (ov), vagina (va), and resorbens vesicle (vr); and male reproductive organs: cirrus (c), prostatic bulb (pb), prostatic vesicle (pv), seminal vesicle (sv), and vasa deferentia (vd) **C** line drawing of cirrus in different focusing planes, showing the sclerites portion of the introvert (sp), and proximal limit of the introvert (arrows). Scale bars: 200 µm (**A**); 100 µm (**B**); 20 μm (**C**).

**Figure 3.**
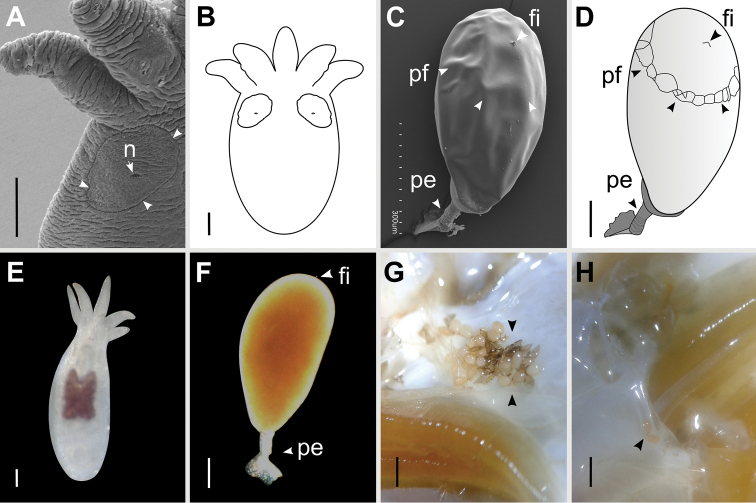
*Temnocephala ivandarioi* sp. nov. details of epidermal excretory syncytial plates (DLSPs) and eggs **A** antero-lateral area observed with SEM showing leftmost tentacle and left DLSP, arrow showing contour and position of excretory pores (n) **B** line drawing of entire specimen showing the DLSP_s_**C** egg observed with SEM showing the filament (fi), peduncle (pe), and plane of fracture of the operculum (pf) **D** line drawing of a whole egg showing the oblique fracture plane to the longitudinal axis of the egg **E** live adult specimen showing red eyespot pigment **F** unhatched egg showing the filament (fi) **G, H** live eggs deposited on branchial chambers of *V. serrata*. Scale bars: 100 μm (**A–F**); 1mm (**G, H**).

***External characteristics.*** Body (without tentacles) 1.36–2.26 mm (1.75 ± 0.25) long by 1.18–1.56 mm (1.36 ± 0.11) wide; adhesive disk ventral, subterminal 280–520 (370 ± 82) long by 320–520 (400 ± 40) wide (Figs 1A–C, 2A); eyespots with red pigmentation (observations made on live specimens; Fig. 3E). DLSPs small, elliptical-shaped (Fig. 3A, B), 167 long by 141 wide (*N* = 2); excretory pore “subcentral” in the DSLP, displaced towards the internal limit (Fig. 3A); length ratio of DLSPs:total body length, without tentacles, 1.0:10.7.

***Alimentary system.*** Mouth surrounded by a large muscular sphincter 200–280 (220 ± 26) long by 210–310 (248 ± 32) wide; pharynx 330–620 (417 ± 56) long by 450–620 (511 ± 52) wide; intestine saccular, without septations (Fig. 2A).

***Glands.*** Rhabditogenic glands forming bunches in the lateral fields of the body extending from the pharynx to the middle level of the adhesive disk. Haswell cells in front of the eyespots and the brain. Disk glands between the adhesive disk and the genital complex (Fig. 2A).

**Figure 4.**
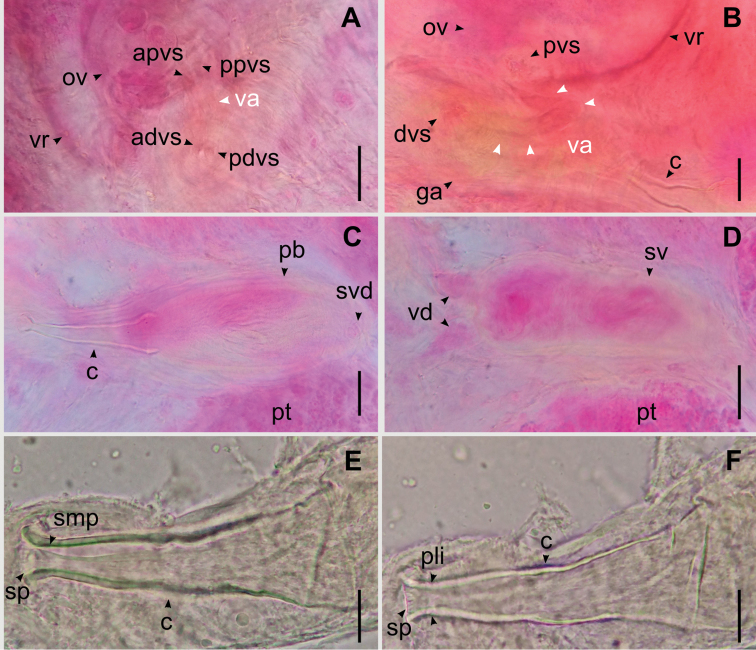
Details of the reproductive system of *Temnocephala ivandarioi* sp. nov. **A, B** partial female reproductive system, showing: anterior portion of the distal vaginal sphincter (advs), anterior portion of the proximal vaginal sphincter (apvs), distal vaginal sphincter (dvs), genital atrium (ga), posterior portion of the distal vaginal sphincter (pdvs), posterior portion of the proximal vaginal sphincter (ppvs), proximal vaginal sphincter (pvs), ovary (ov), vagina (va), and vesicula resorbens (vr) **C, D** partial male reproductive system, showing: cirrus (c), prostatic bulb (pb), seminal vesicle duct (svd), seminal vesicle (sv), and vasa deferentia (vd) **E, F** cirrus introvert observed in different focusing planes, view of the circle of sclerites (sp) in the distal portion of the introvert and the smooth portion (smp) in the proximal limit of the introvert (pli). Scale bars: 50 μm (**A–D**); 20 μm (**E, F)**.

**Female.** Ovary ventral to the resorbens vesicle 57–100 (83 ± 13; *N* = 7), long by 60–145 (105 ± 26; *N* = 7) wide. Vagina elongated with strong muscular wall, connects to the genital atrium dorsally, 75–180 (125 ± 37; *N* = 4) long by 16–30 (23 ± 4; *N* = 6) wide with a widening of the distal portion; proximal vaginal sphincter symmetrical 16–34 (23 ± 6; *N* = 6) and distal vaginal sphincter symmetrical (16–20; *N* = 2) (Figs 2B, 4A, B). Resorbens vesicle ovoid 110–180 (134 ± 24, *N* = 9) long by 172–212 (194 ± 16; *N* = 9) wide. Vitellarium arborescent and thin (Fig. 2A). Eggs 557–638 (585 ± 37; *N* = 4) long by 302–331 (312 ± 13; *N* = 4) wide; filament small, subapical or apical (Fig. 3C, D, F); peduncles 146–243 (341 ± 97); the plane of fracture is oblique with respect to the longitudinal axis of the egg (Fig. 3C, D). Eggs deposited on branchial chambers of host (Fig. 3G, H).

**Male.** Two pairs of testes, medium-sized, usually rounded, slightly oblique, anterior testes 180–310 (231 ± 34) long by 120–320 (220 ± 48) wide; posterior testes 200–400 (260 ± 60) long by 110–360 (254 ± 65) wide (Fig. 2A). Seminal vesicle dorsal and anterolateral to the prostatic bulb (Figs 2B, 4D), 52–137 (92 ± 27) long by 82–237 (168 ± 47) wide, wall 8.6 thick. Prostatic bulb 70–107 (91 ± 14) long by 155–240 (191± 30) wide (Figs 2B, 4C). Cirrus small-sized, 120–147 (129 ± 8) long; shaft cone-shaped, slightly curved up, with maximum width at base 40–47 (44 ± 2); introvert cone-shaped, not oblique, not curved, with a circle of sclerites (range 18–20) in the distal portion followed by a smooth portion without spines or ridges, 7.5–15 (10 ± 3; *N* = 8) long, with maximum width 15–22 (18 ± 3; *N* = 10) at level of the distal portion (Figs 2C, 4C, E, F, 6A). Ratio between total body length, without tentacles:total length of cirrus 14:1; ratio between total length of cirrus:width of shaft´s base 3:1; ratio between total length of cirrus:total length of introvert 13:1.

##### Etymology.

The new species is dedicated to Dr. Iván Darío Vélez Bernal for his outstanding contributions to the study of helminthology and the understanding of tropical diseases in Colombia.

##### Discussion.

Temnocephalida is a monophyletic group within the Platyhelminthes included in Lymnotyphloplanida, which is part of the Dalytyphloplanida clade, a major group of Rhabdocoela (Van Steenkiste et al. 2013). Temnocephalidae Monticelli, 1899 is the most diverse family of the Temnocephalida. Its members are distributed around Australia and the Neotropics (Martínez-Aquino et al. 2014); the type genus of Temnocephalidae, *Temnocephala* Blanchard, 1849, is exclusive to the Neotropics. Autapomorphies of the *Temnocephala* include red-pigmented eyespots, four epidermal syncytial plates, and excretory pores enclosed within the boundaries of the DLSPs (Damborenea and Cannon 2001). Major hosts to the members of the *Temnocephala* are chelonians, molluscs, insects, and crustaceans, each hosting a particular assemblage of *Temnocephala* species. Particular host families are also specific for particular *Temnocephala* species (Martínez-Aquino et al. 2014).

Taxonomy of temnocephalans is based on morphology of adult specimens with emphasis on the reproductive system. The structure of the cirrus is the trait of greatest taxonomic value (Damborenea 1991, Damborenea and Cannon 2001, Sewell et al. 2007, Garcés et al. 2013). Other traits important for species differentiation include composition of the female reproductive complex, eggs deposit areas in the host, and the shape of the DLSPs (Damborenea and Brusa 2008, Amato et al. 2010, Volonterio 2010, Seixas et al. 2011, 2015, 2018).

Nine species of *Temnocephala* are known for their association with crabs of the Trichodactylidae family. Of these, *T. ivandarioi* sp. nov., *T. longivaginata* Seixas, Amato & Amato, 2011, and *T. lutzi* Monticelli, 1913 (Amato et al. 2005) present a similar-sized cirri and have the Amazon River basin as a biogeographical connection. *Temnocephala longivaginata* and *T. ivandarioi* sp. nov. are most similar to each other in the length of the vagina and the presence of sclerites in the distal portion of the cirrus.

*Temnocephala ivandarioi* sp. nov. can be distinguished by the combination of the following features: cirrus with a circle of small sclerites (range 18–20) in the distal portion of the introvert, without spines or ridges in the inner wall of the introvert (Fig. 2C). The ovary lies ventral to vesicle resorbens followed by an elongated vagina with two vaginal sphincters similar in size, one symmetric and proximal, and one symmetric and distal; the vagina connects to the genital atrium dorsally. The seminal vesicle is located anterolateral to the prostatic bulb. The DLSPs are small and ‘elliptical-shape’, with a partially sinuous contour.

On an ecological-level *T. ivandarioi* sp. nov., *T. longivaginata*, and *T. lutzi* inhabit the branchial chambers of trichodactylid crabs from the middle basin and lower basin of the Amazon River (Leticia, Amazonas, Colombia; Peixe-Boi, Pará State; Rio Amapá, Amapá State, northern Brazil, respectively). *Temnocephala ivandarioi* sp. nov. is the third species described from Colombia, and therefore *V. serrata* is registered as a new trichodactylid host for neotropical temnocephalans. *Valdivia serrata* is widely distributed throughout the Orinoco and Amazon River basins in Venezuela, the islands of Trinidad and Tobago, the Guianas, Colombia, Brazil, Peru and Bolivia (Cumberlidge 2008). In Colombia this species is found in the eastern region of the country (Amazonas, Arauca, Caqueta, Meta, Putumayo, and Vichada Departments) in the Putumayo and Maqueta rivers that drain into the Amazon River, and the Guaviare, Meta, and Arauca rivers that drain into the Orinoco River (Campos 2005, 2014). It is likely that *T. ivandarioi* sp. nov., *T. longivaginata*, and *T. lutzi* are closely related due to their morphological similarities and geographical proximity. The implementation of molecular studies will reveal the phylogenetic relationships between the different species of *Temnocephala* in the Neotropics.

In Colombia more than 132 species of decapod crustaceans have been recorded (Campos 2014), while only two associated species of temnocephalans have been reported to date: *T. icononcensis* (Arias-Pineda et al. 2015) and *T. ivandarioi* sp. nov. The great diversity of these potential hosts (Campos 2014) suggests that most temnocephalans remain undescribed.

##### Comparative notes.

**Figure 5.**
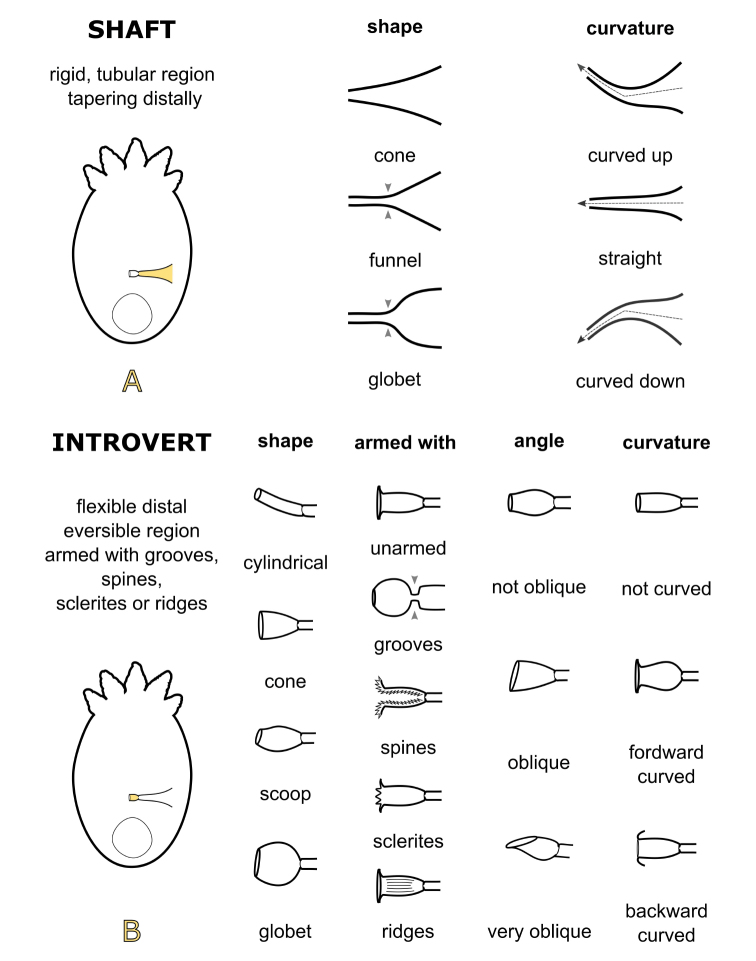
Diagrams showing the terminology used to describe the cirrus of the species of *Temnocephala* (terminology updated from Sewell et al. (2007), Seixas et al. (2011), Garcés et al. (2013), and Ponce de León et al. (2015); diagrams modified from Sewell et al. 2007: 205, fig. 2).

**Figure 6.**
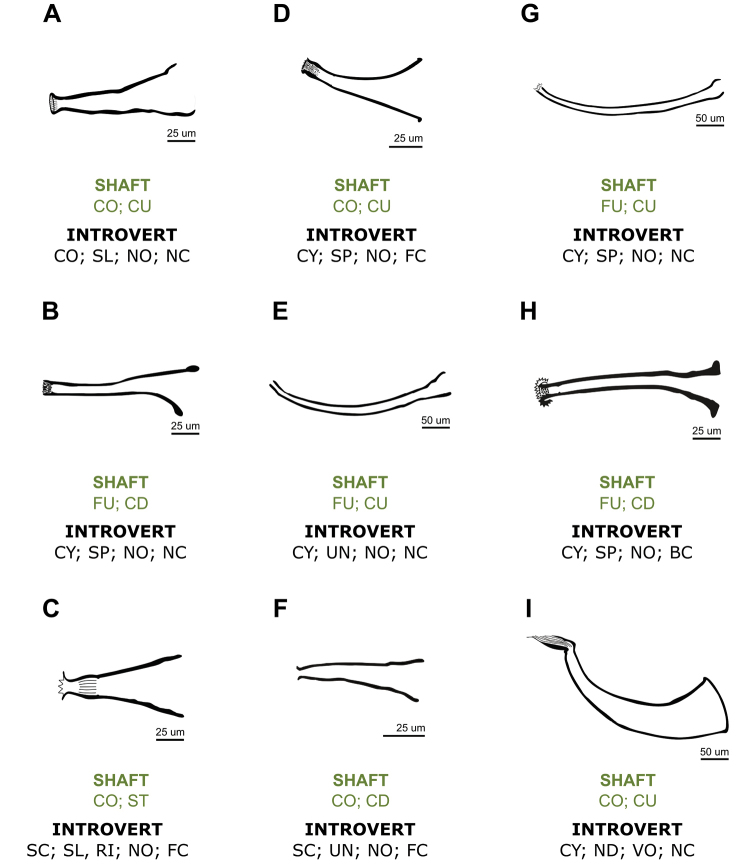
Diagrams of the cirrus of the species of *Temnocephala* associated with trichodactylid crabs. Terminology based on the cirrus structure (see comparative notes and Fig. 5). **A***Temnocephala ivandarioi* sp. nov. (present study) **B***Temnocephala lanei* Pereira & Cuocolo, 1941 **C***Temnocephala longivaginata* Seixas, Amato & Amato, 2011 **D***Temnocephala lutzi* Monticelli, 1913 (Amato et al. 2005) **E***Temnocephala microdactyla* Monticelli, 1903 **F***Temnocephala pignalberiae* Dioni, 1967 (Amato et al. 2010) **G***Temnocephala santafesina* Dioni, 1967 **H***Temnocephala trapeziformis* Amato, Amato & Seixas, 2006 **I***Temnocephala travassosfilhoi* Pereira & Cuocolo, 1941. Key: **Shaft** [**shape: CO**–cone; **FU**–funnel]; [**curvature: CU**–curved up; **ST**–straight; **CD**–curved down]. **Introvert** [**shape: CY**–cylindrical; **CO**–cone; **SC**–scoop]; [**armed with: UN**–unarmed; **SP**–spines; **SL**–sclerites; **RI**–ridges; **ND**–not described]; [**angle: NO**–not oblique; **VO**–very oblique]; [**curvature: NC**–not curved; **FC**–forward curved; **BC**–backward curved].

The cirrus is the only rigid structure and therefore of constant general morphology in juveniles and adults (except for small intraspecific variations) for each species. The morphology of the cirrus constitutes one of the few characters used and is the most valuable taxonomic character for species identification (Seixas et al. 2015b). In the present study, terminology describing the temnocephalan cirrus is updated for neotropical species (Fig. 5), according to Sewell et al. (2007), Seixas et al. (2011), Garcés et al. (2013) and Ponce de León et al. (2015). The cirrus of the species of *Temnocephala* described from trichodactylid crabs (Fig. 6) are compared based on this terminology.

The cirrus is defined as the entire sclerotised male copulatory organ comprised of a ‘shaft’ (rigid, tubular region tapering distally; Fig. 5A) and an ‘introvert’ (flexible distal eversible region armed with grooves, spines, sclerites or ridges, Fig. 5B) (modified from Sewell et al. 2007). Furthermore, the degree of shaft curvature is a reliable taxonomic characteristic of neotropical temnocephalans (Garcés et al. 2013).

The shape of the shaft may be described as a ‘funnel’, ‘goblet’, or ‘cone’. Funnel- or goblet-shaped shafts have a wide proximal region which tapers rapidly into a narrow tubular distal region (Sewell et al. 2007). The cirrus may be more or less curved, and it may be described as ‘curved up’, ‘straight’, or ‘curved down’. Similarly, the position of the cirrus with respect to the body may be described as ‘towards the forebody’, ‘horizontal’, or ‘towards the hindbody’ (modified from Garcés et al. 2013). The position of the cirrus can or may not depend on the cirrus curvature i.e. cirrus ‘curved up’ directed ‘towards the forebody’, but cirrus ‘straight’ are directed towards the ‘forebody’, ‘horizontal’ or ‘towards the hindbody’. The cirrus position can be examined only from a complete diagram of the temnocephalan.

The introvert shape can be described as ‘cylindrical’, ‘cone’; ‘scoop’, or ‘goblet’. Scoop- or goblet-shaped introvert have a wide middle region, which tapers into a narrow distal region. In addition, the introvert may be ‘unarmed’, armed with ‘grooves’ in the proximal limit of the introvert, or armed with ‘spines’, ‘sclerites’, and ‘ridges’ in the inner wall of the introvert. The distal opening of the introvert may be at right angles with respect to the proximal limit of the introvert i.e. ‘not oblique’, ‘oblique’, or ‘very oblique’ (modified from Sewell et al. 2007). Additionally, the distal region of the introvert may be curved (with or without spines, sclerites, or ridges), and described as ‘forward curved’, ‘straight’ or ‘backward curved’ (described as with non-spined region or without non-spined region by Sewell et al. 2007).

The morphology of the cirrus is necessary for species identification and should be clearly described based on the terminology proposed in the present study. This new terminology can be applied to species of neotropical temnocephalans described to date.

## Supplementary Material

XML Treatment for Temnocephala ivandarioi
